# Preliminary study of the UL55 gene based on infectious Chinese virulent duck enteritis virus bacterial artificial chromosome clone

**DOI:** 10.1186/s12985-017-0748-y

**Published:** 2017-04-13

**Authors:** Ying Wu, Yangguang Li, Mingshu Wang, Kunfeng Sun, Renyong Jia, Shun Chen, Dekang Zhu, Mafeng Liu, Qiao Yang, Xinxin Zhao, Xiaoyue Chen, Anchun Cheng

**Affiliations:** 1 Institute of Preventive Veterinary Medicine, Sichuan Agricultural University, Chengdu, Sichuan 611130 China; 2 Key Laboratory of Animal Diseases and Human Health of Sichuan Province, Chengdu, Sichuan 611130 China; 3 Avian Diseases Research Center, College of Veterinary Medicine, Sichuan Agricultural University, Chengdu, Sichuan 611130 China

**Keywords:** Duck enteritis virus, Bacteria artificial chromosome, Chinese virulent strain, UL55

## Abstract

**Background:**

Lethal Duck Enteritis Virus (DEV) infection can cause high morbidity and mortality of many species of waterfowl within the order Anseriformes. However, little is known about the function of viral genes including the conserved UL55 gene among alpha herpes virus due to the obstacles in maintenance and manipulation of DEV genome in host cells.

**Methods:**

In this paper, we constructed an infectious bacteria artificial chromosome (BAC) clone of the lethal clinical isolate duck enteritis virus Chinese virulent strain (DEV CHv) by inserting a transfer vector containing BAC mini-F sequence and selection marker EGFP into UL23 gene using homologous recombination. UL55 deletion and its revertant mutant were generated by two-step RED recombination in *E. coli* on basis of rescued recombinant virus. The function of UL55 gene in DEV replication and its effect on distribution of UL26.5 protein were carried out by growth characteristics and co-localization analysis.

**Results:**

The complete genome of DEV CHv can be stably maintained in E. coli as a BAC clone and reconstituted again in DEF cells. The generated UL55 deletion mutant based on DEV CHv-BAC-G displayed similar growth curves, plaque morphology and virus titer of its parental virus in infected Duck Embryo Fibroblast (DEF) cells. Immunofluorescence assay indicated that the loss of UL55 gene do not affect the distribution of UL26.5 protein in intracellular. These data also suggest infectious BAC clone of DEV CHv will facilitate the gene function studies of DEV genome.

**Conclusions:**

We have successfully developed an infectious BAC clone of lethal clinical isolate DEV CHv for the first time. The generated UL55 gene mutant based on that demonstrated this platform would be a very useful tool for functional study of DEV genes. We found the least known DEV UL55 is dispensable for virus replication and UL26.5 distribution, and it could be a very promise candidate locus for developing bivalent vaccine. Experiment are now in progress for testifying the possibility of UL55 gene locus as an exogenous gene insertion site for developing DEV vectored vaccine.

**Electronic supplementary material:**

The online version of this article (doi:10.1186/s12985-017-0748-y) contains supplementary material, which is available to authorized users.

## Background

Duck Viral Enteritis (DVE), also known as Duck Plague (DP), is an acute, febrile, septic and contagious disease of ducks, geese, swans and many other species of birds within the order Anseriformes caused by Duck Enteritis Virus (DEV) [1]. The morbidity and mortality of infected young ducklings or unprotected ducks reaches up to 100%, resulting in huge economic losses of domestic and wild waterfowls worldwide [2–4]. In addition, after primary infection, the viruses persist in their host for life. They hide from the immune system in a latent state in which their genome is almost dormant. Eventual episodes of reactivation allow them to infect naive individuals, causing a long-term of prevalence in the high density duck raising farms after DVE outbreak [5]. Therefore, even live attenuated DEV vaccines have been used in prevention and control of this lethal disease since 1960s, the DEV infection is not completely prevented [6–10]. Thus, investigation of DEV gene functions and pathogenesis will be an intelligent policy to novel vaccine development and disease controlling.

According to the reports, studying the resulting variations in phenotype of virus mutants can be very useful for understanding the fundamental information of virus gene function and pathogenesis, which provide theoretical basis for development of novel vaccines and chemotherapeutics [11]. A bacteria artificial chromosome (BAC) can take up the complete genome of a herpes virus as an infectious clone for randomly mutagenesis without requirement of restriction sites or cloning steps in E. coli have been developed for herpes virus mutagenesis and reconstitution which extensively facilitated functional studies of viral genes [11]. After the first case of infectious herpes virus BAC clone has been successfully applied in murine cytomegalovirus (MCMV) in 1997 [12], the strategy of cloning full length of herpesvirus genome as a BAC has been adopted for herpes virus. In most recent years, infectious BAC clone has been gradually applied in DEV study. Wang et al. firstly generated an infectious BAC clone of the European virulent DEV strain 2085 and expressed hemagglutinin (H5) of high pathogenicity H5N1 avian influenza virus (AIV) based on that. The insertion of BAC components into DEV 2085 genome UL44 (gC) gene caused disruption of gC function [13]. In China, full length of DEV vaccine strains VAC and C-KCE were extensively cloned into BAC to generate bivalent vaccines for protection of ducks against DEV and other poultry pathogens. DEV glycoprotein gC, junctions of UL26 and gB, regions between UL15B and UL18 gene, SORF3 and US2 junctions were selected for insertion of BAC Mini-F sequence [6, 14–18]. From these literatures, we found that DEV infectious BAC clones were predominantly considered as an ideal vector for constructing live bivalent vaccines. However, rarely reports focused on the functional characterization and pathogenesis of lethal DEV that will lead to high morbidity and mortality of infected ducks. Although bivalent live vaccine can protect ducks against DEV and other causative pathogens infection at one time, but lacking of molecular background of the DEV carrier and integration of two pathogens may lead to potential security risks in a relatively long period which we cannot predicted. Therefore, constructing a platform that can be used for functional characterization and pathogenesis investigation of virulent clinical isolate DEV will benefit the long-term development of novel vaccines and disease control.

To our knowledge, previously studies about DEV almost focused on the epidemiology and prevention of this disease. Limited molecular biology data are available regarding DEV genome and its encoding proteins except some significant genes related to virus infection, replication and antiviral effect. As one of the least known ORFs among total 78 genes of DEV genome, homologues of the DEV UL55 protein (pUL55) are encoded only among alpha herpes viruses [19]. Reports about herpes simplex virus 2 (HSV-2) UL55 protein revealed that the product of HSV-2 UL55 gene may play an accessory role in virion assembly or maturation and has some relationship with UL26.5 distribution [20], but the corresponding homologue gene of EHV-1 was supposed to mediate persistent infection [21]. Pre-existing data suggested that the HSV-1 UL55 gene was not critical for intraperitoneal virulence or establishment of latent infection [22], but subsequent documents suggested this dispensable gene was thought to be important for virus growth and spread in the natural host. However, the characteristics of DEV UL55 gene in DEF cells remains unclear due to limited data. Thus, carrying out some functional studies of UL55 gene will provide some data for further studies of DEV pathogenesis.

In this study, we firstly constructed an infectious BAC clone of the Chinese virulent DEV strain (DEV CHv) to generate a platform that can be used for functional characterization of DEV genes in DEF cells. The UL23 gene of DEV, which has been demonstrated nonessential for DEV replication, was used for inserting BAC mini-F sequence and screening marker EGFP [23–26]. The functional studies of DEV CHv UL55 gene in DEF cells was carried out based on this newly established platform by using two-step RED recombination for generation of UL55 deletion and revertant mutants in *E. coli*. The data yielded by this study demonstrated that UL55 gene is nonessential for virus replication and has no effect on distribution of UL26.5 protein in infected cells. As a result, we can conclude that this DEV BAC system will facilitate the studying of the biology and gene functions of DEV field strain. Moreover, the nonessential UL55 gene could be a candidate locus for developing bivalent vaccine after attenuation.

## Methods

### Cells, virues, plasmids and antibodies

The DEV CHv strain (Accession NO. JQ647509) was used for the construction of infectious BAC clone. Duck embryo fibroblast (DEF) cultures prepared from 10-day-old cherry valley duck embryos were used for the propagation of DEV CHv and its derived mutants, which was maintained in minimal essential medium (MEM, Gibico) supplemented with 100 U/mL penicillin, 100 mg/mL streptomycin (1% P/S) and 10% new calf serum (NCS,Gibico) at 37 °C under a 5% CO_2_ atmosphere. The help plasmids pKD46, pKD4 and Pcp20 were kindly donated by Prof. Kelly T. Hughes (University of Utah), while the plasmid pBeloBAC11 and E. coli strain DH10B was supplied by Yunfeng Wang (Harbin veterinary research institute, Chinese Academy of Agricultural Science). Our lab prepared the antibodies against UL55 protein and UL26.5 protein by immune healthy rabbits.

### Constrution of transfer vector pUC18/EGFP-TKAB-BAC11

The plasmid pBeloBAC11 containing the mini-F sequence was used for cloning of the DEV CHv complete genome. It was carried out broadly on the same principle used for the construction of the BAC clones of MDV-1, HVT, VZV, BoHV-1 and EHV-1 etc. by inserting the bacterial mini-F sequence and the enhanced green fluorescent protein (EGFP) into UL23 (TK) gene of DEV CHv through homologous recombination. The strategy for constructing the transfer vector containing the essential functional components (mini-F) of BAC, EGFP and UL23 homologous sequence to facilitate recombination was shown in Additional file 1: Figure S1. The generated transfer vector pUC18/EGFP-TKAB-BAC11 harboring the homologous regions of 1357 bp on the left and 1039 bp on the right flanking regions of TK insertion site, mini-F sequence and a cellular screening marker EGFP.

### Constrution of BAC clone of DEV CHv

The cloning of DEV CHv complete genome into BAC plasmid was performed by homologous recombination in DEF cells (Additional file 2: Figure S2 a, b). To the details, approximately 2.5 μg pUC18/EGFP-TKAB-BAC11 was transfected into freshly seeded primary DEF cells infected with DEV CHv strain by using Lipofectamine 3000 (Invitrogen). Meanwhile, the DEV CHv infected cells and mock-infected cells transfected with pEGFP-∆MCS were did in parallel as controls. When the green fluorescence plaques appeared, several rounds of EGFP positive plaques selection were performed for recombinant virus purification and enrichment. Genomic DNA of the cultures was obtained at the next step by sodium dodecyl sulfate (SDS)-proteinase K extraction as described earlier [27] for identification of the purified BAC-recombinant DEV CHv by PCR using the primers listed in Table 1. 100 ng identified BAC-recombinant DEV CHv DNA was electroporated (1.8 KV, 200 Ω, 25 μF) into E. coli DH10B cells using a Bio-Rad E. coli Pulser with 0.1 cm cuvettes. The colonies grown on chloramphenicol plate were detected by PCR to confirm the presence of EGFP, essential functional components of BAC and some important genes related to replication, virulence and structure of DEV CHv virions. Furthermore, plasmid extracted from the PCR identified chloramphenicol-resistant BAC clones was digested with *EcoR* I for RFLP analysis, the correct plasmid was named pBAC-DEV and further identified by sequencing (Invitrogen).

**Table 1:** Primers used in this paper

NO.	Primers	Sequence (5’-3’)	Product
1	TKA-HOMO-for	gaattcatgcttgccatcataaccgtattctc	TK left homology arm
	TKA-HOMO-rev	tctagaataacttcgtataatgtatgctatacgaagttatcacctcgagcttttctttcctgtg	
2	TKB-HOMO-for	gcatgcacatagcaacaactgacgcaaaagc	TK right homology arm
	TKB-HOMO-rev	aagctttcccagaaagctcgcctaggtcctc	
3	EGFP-for	tctagatagttattaatagtaatcaattacg	EGFP
	EGFP-rev	gtcgacatgcagtgaaaaaaatgct	
4	sopB-for	attcgttaattgcgcgcgtagg	sopB
	sopB-rev	gaatattcaggccagttatgct	
5	repA-for	catggcggaaacagcggttatc	repA
	repA-rev	atgtatgagaggcgcattggag	
6	TK-for	cgcggatcccactgaatgtcactgc	TK
	TK-rev	cccaagctttcaattaattgtcatctcggt	
7	ΔUL55-KanR-for	gaaaggcggttggaataagaggaacgaggcggtagacgtgaccgacaacagtgtaggctggagctgcttc	KanR gene flanked by homology arms of UL55
	ΔUL55-KanR-rev	tttcttatggttttaataaaacgctttattacattgtagtgtaacaagaccatatgaatatcctccttag	
8	ΔUL55/ΔUL55R-for	tgcaaattagtgggaggtacg	ΔUL55/ΔUL55R identification product
	ΔUL55/ΔUL55R-rev	cccaaataccctgttagtagctt	
9	ΔUL55R-UL55-for	atggccgacgcgaaggcggt	UL55 fragment with left homology arm of UL55
	ΔUL55R-UL55-rev	gaagcagctccagcctacac*tcatacattagctttgtg*	
10	ΔUL55R-KanR-for	*cacaaagctaatgtatga*gtgtaggctggagctgcttc	UL55 fragment with right homology arm of UL55
	ΔUL55R-KanR-rev	tttcttatggttttaataaaacgctttattacattgtagtgtaacaagaccatatgaatatcctccttag	

Italic: Complementary sequence for overlap PCR

### Reconstitution of infectious virus from pBAC-DEV clone

For the rescue of infectious recombinant virus in host cells, 2.5 μg freshly prepared pBAC-DEV DNA by QIAGEN Midi Kit was transfected into DEF cells using Lipofectamine 3000 (Invitrogen). Cells transfected with or without pEGFP-ΔMCS were taken as controls. Cells with green fluorescence CPE were harvested and passaged for enrichment of infectious virus production. The infectious recombinant virus named DEV CHv-BAC-G will be obtained until complete cytopathic effect had been developed.

For determination of the reconstitute DEV CHv-BAC-G, PCR was performed firstly for the identification of DEV CHv gene and the functional components of BAC. A rabbit polyclonal antibody against DEV UL23 gene was used to detect the expression of TK protein in DEV CHv-BAC-G infected DEF cells by indirect immunofluorescence analysis (IFA). Mock and wild type DEV CHv infected DEF cells were used as controls to demonstrate the specificity of anti-TK for DEV CHv. In IFA, DEF cells were grown on glass coverslips and infected with either wild type DEV CHv or reconstitute DEV CHv-BAC-G at a multiplicity of infection (MOI) of 0.02, 200 μl anti-TK IgG at a 1:50 dilution was used as first antibody. The corresponding pDEV-BAC that can produce green fluorescence plaques was isolated for sequencing.

### Chracterization of the rescued virus in vitro

Growth curve analyses were performed to compare the growth kinetics of the wild type DEV CHv with that of the reconstitute virus DEV CHv-BAC-G. Briefly, DEF monolayers grown on 24-well plates were infected with 0.02 MOI of wild type and reconstitute virus. Supernatants containing virus were harvested directly at 7, 12, 24, 36, 48, 72 h post infection (h.p.i), while the cells and mixture of cultures were harvested at the indicated time points by treating with trypsin. The amount of infectious wild type and reconstitute virus in the harvested supernatants and cells were determined by Reed-Muench assay as previously studies described [28]. Growth curves assay was performed in triplicate in three independent experiments.

To determine plaque areas of DEV CHv and DEV CHv-BAC-G, DEF cells were infected at an MOI of 0.02. The inoculated viruses were discarded at 1 h post infection before adding 500 μl MEM containing 0.5% methylcellulose and 10% NCS. After 3–5 days, the methylcellulose in each well was replaced with 500 μl pre-cold 4% paraformaldehyde for cells immobilization. Cells were then washed and stained with 500 μl crystal violet for 5 min. Thorough washing were performed subsequently to remove the staining solution. Plaques were photographed, and the average plaque areas were determined using the Image J software (http://rsb.info.nih.gov/ij/). Values of DEV CHv-BAC-G were calculated and compared to wild type DEV CHv plaque areas, which were set to 100%. T-tests were used to assess the significance of the results (*P* < 0.05). Average percentages of plaque diameters and standard deviations were determined from at least three independent experiments.

Transmission electron microscopy of the 0.1 MOI DEV CHv-BAC-G infected DEF cells were performed according to previously reports [29]. Briefly, cells were washed with PBS at 36 h.p.i, and fixed with 2.5% glutaraldehyde at 4 °C for 2 h. After that, the fixed adherent cells were collected by scraping from the flasks and centrifuged at 40,000 rpm/min for 2 h. Then the pellets were mixed with 2% low melting-temperature agarose at 37 °C, and centrifuged at 6000 rpm/min for 10 min. Samples were post-fixed in 1.0% osmium tetroxide. After a stepwise dehydration in acetone, samples were embedded in epoxy resin 618 and polymerized at 80 °C for 72 h. Then, 50 nm ultra-thin sections were prepared with an LKB ultra tome (LKB Instruments, Inc., Rockville, MD), collected on grids, and stained with uranyl acetate and lead citrate for subsequent examination with the Hitachi H-600-A2 transmission electron microscope at an accelerating voltage of 75 kV. Images were recorded on Kodak electron microscope film and compared to that of DEV CHv virions.

### Generation and characterization of UL55 deletion and its revertant mutant based on established recombinant DEV CHv-BAC-G

Cloning of wild type DEV CHv as infectious BAC clones facilitates easy manipulation of their genomes. In order to test the amenability of pBAC-DEV clone to genomic modification for DEV CHv gene study, we constructed a UL55 gene deletion and its revertant mutants by two-step RED recombination [30]. In brief, 100 ng PCR product of kanR cassette flanked by the FRT sites and homology arms of UL55 gene was electroporated into DH10B cells containing pBAC-DEV clone and pKD46 to complete the first round of recombination induced by 100 mM L-arabinose for expression of RED recombination related proteins exo, beta, gam in pKD46. Kanamycin resistant colonies were isolated on LB/Cm/Kana plates. In the second round of RED recombination, the temperature sensitive plasmid Pcp20 was transformed into DH10B cells containing kanR colonies of the 1st recombination to trigger the excising of kanR FRT cassette. The induced Pcp20 can be removed at 42 °C and the positive colonies identified by PCR and sequencing was named as DEV CHv-BAC-GΔUL55 (Additional file 2: Figure S2 b,c).

The construction of DEV CHv-BAC-GΔUL55 revertant following the same mutagenesis protocol. In the 1st round of RED recombination, overlapping PCR product of complete UL55 gene and kanamycin resistance gene flanked by the homology arms of UL55 deletion region was inserted into the UL55 mutation locus (Additional file 2: Figure S2 c,d). The revertant mutant DEV CHv-BAC-GΔUL55R was obtained after excising of KanR in the 2nd round of RED recombination (Additional file 2: Figure S2 d). Infectivity of the DEV CHv-BAC-GΔUL55 and DEV CHv-BAC-GΔUL55R mutants were examined by transfection of them into DEF cells as their parental strain DEV CHv-BAC-G did. PCR, IFA and RFLP analysis were carried out as we described above to confirm the presence of DEV CHv genome and BAC functional components, and the mutation of UL55 region. Growth characteristics of DEV CHv-BAC-GΔUL55 and DEV CHv-BAC-GΔUL55R were compared to their parental virus DEV CHv-BAC-G as we previously described to determine the function of UL55 in DEV replication.

### The effect of UL55 protein on the distribution of UL26.5 protein in infected DEF cells

To investigate the intracellular distribution correlation of UL55 and UL26.5 proteins, DEF cells infected with 0.02 MOI wild type DEV CHv and recombinant viruses were collected for IFA in two independent experiments. In the first experiment, DEV CHv infected DEF cells were collected at 60 h.p.i. and fixed with 4% paraformaldehyde. After washing step, the fixed cells were incubated with 1:50 diluted mouse anti-UL55 or 1:100 rabbit anti-UL26.5 IgG prior to addition of 1:100 diluted TRITC-conjugated Goat anti-mouse IgG or FITC-conjugated Goat anti-rabbit IgG for the single distribution analysis of UL55 and UL26.5 encoded proteins in host cells. For co-localization analysis, 1:50 diluted mouse anti-UL55 and 1:100 rabbit anti-UL26.5 IgG were added together into harvested DEF cells as primary antibody while the mixture of 1:100 diluted TRITC-conjugated Goat anti-mouse IgG and FITC-conjugated Goat anti-rabbit IgG were added as secondary antibody. DEF cells infected with 0.02 MOI parental DEV CHv-BAC-G and its derived mutants DEV CHv-BAC-GΔUL55 and DEV CHv-BAC-GΔUL55R were collected and fixed as mentioned above to demonstrate the contribution of UL55 gene to UL26.5 protein distribution. 1:100 diluted rabbit anti-UL26.5 IgG and TRITC-conjugated Goat anti-mouse IgG were used as the first and secondary antibodies for IFA Fluorescence of samples above were inspected under invert fluorescence microscope.

## Results

### Cloning of the full length of DEV CHv genome into E. coli

**Fig. 1:**
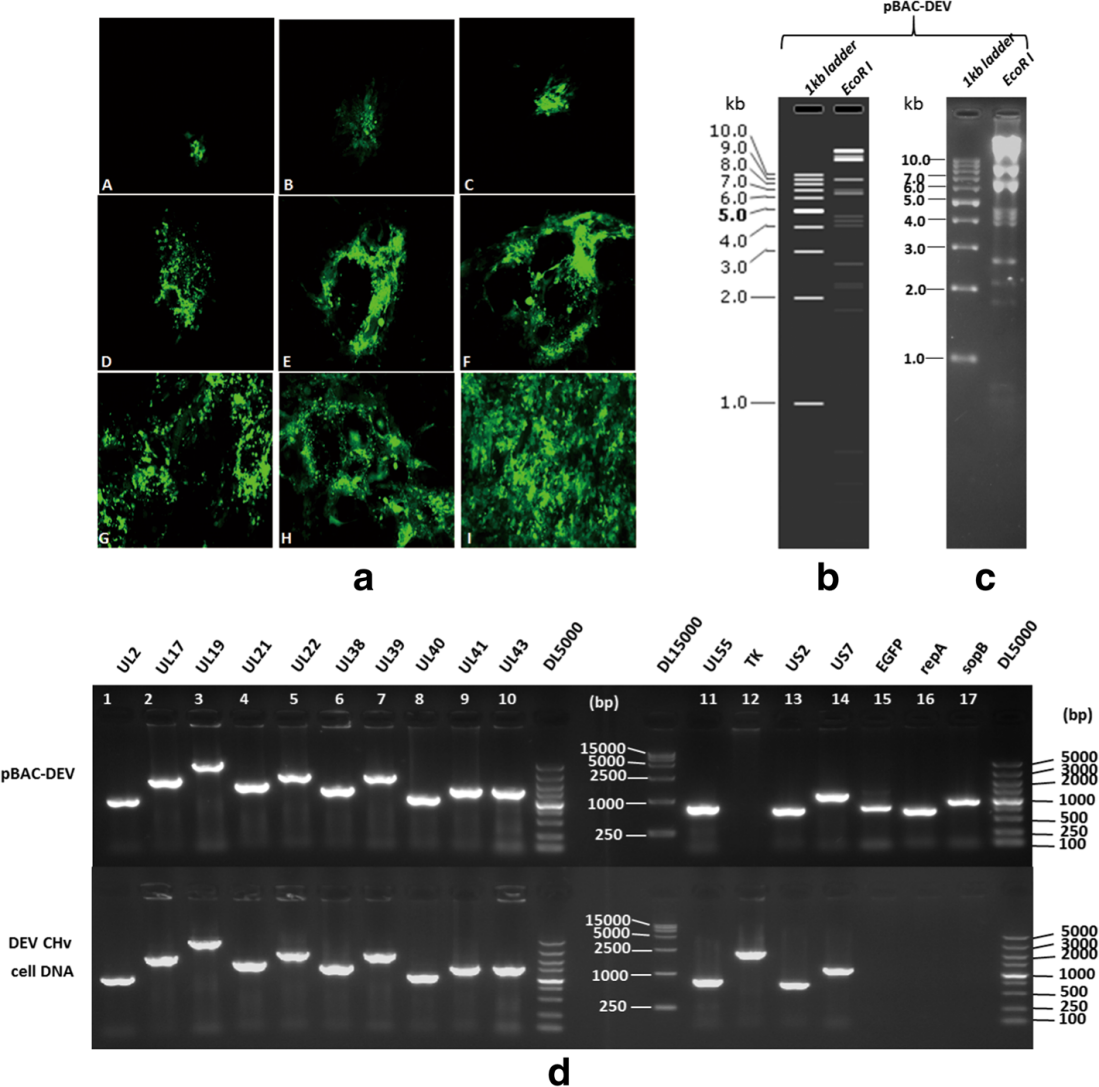
Identification of recombinant DEV CHv BAC colonies. **a** Purification and enrichment of BAC-recombinant virus. *A*: The 3rd passage of BAC-recombinant DEV after infection. *B-I*: Purification and enrichment of BAC-recombinant DEV by eight round of fluorescence plaque selection. **b** Orientation analysis of pBAC-DEV digested b EcoR I. **c** Real Gel analysis of pBAC-DEV digested by EcoR I. **d** Identification of pBAC-DEV. Lane 1–14: PCR product of DEV genes in table 2; Lane 15–17: PCR product of inserting exogenous gene EGFP, repA and sopB

The strategy for cloning and mutagenesis of the DEV CHv genome in *E. coli* was shown in Additional file 1: Figure S1 and Additional file 2: Figure S2. As a result, the sequenced transfer vector pUC18/EGFP-TKAB-BAC11 containing the mini-F sequence of BAC, EGFP and flanked two loxP sites was used for cloning of the complete genome of DEV CHv in pBeloBAC11 by homologous recombination. As shown in Fig. 1a (A), the DEF cells containing pUC18/EGFP-TKAB-BAC11 DNA and wild type DEV CHv developed green fluorescence after 3 passages, indicating the successfully construction of BAC-recombinant virus. After further purification and enrichment of the recombinant virus, most of the BAC-recombinant virus infected cells exhibited green CPE (Fig. 1a (B-I)). PCR was performed subsequently to confirm the purification of BAC-recombinant virus. repA, sopB gene in BAC plasmid and selection marker EGFP gene can be amplified while TK gene is not presented in the genome of BAC-recombinant virus as expected (data not shown).

Next, the circular DNA of BAC-recombinant virus was then isolated from infected cells and electroporated into *E. coli* DH10B cells. Plasmid DNA extracted from the single chloramphenicol resistance colonies named pBAC-DEV were subjected to RFLP analysis (Fig. 1b, c). As a result, the restriction patterns of *EcoR I* digestion products of pBAC-DEV were as same as we predicted (Fig. 1b). Further identification was carried out by PCR to determine the complete of DEV CHv genome and the existence of inserted EGFP and mini-F sequence of BAC. Figure 1d shows the presence of BAC functional components, EGFP gene and some important genes that play key roles in virus replication, structure and virulence in pBAC-DEV genome. Meanwhile, the absence of the corresponding BAC genes in DEV CHv infected cells demonstrated that a bacterial clone containing a BAC with a full-length DEV CHv genome was successfully constructed. Absence of TK gene in pBAC-DEV could be attributed to the insertion of transfer vector in TK gene.

### Reconstitution of infectious virus from the DEV CHv BAC plasmid

**Fig. 2:**
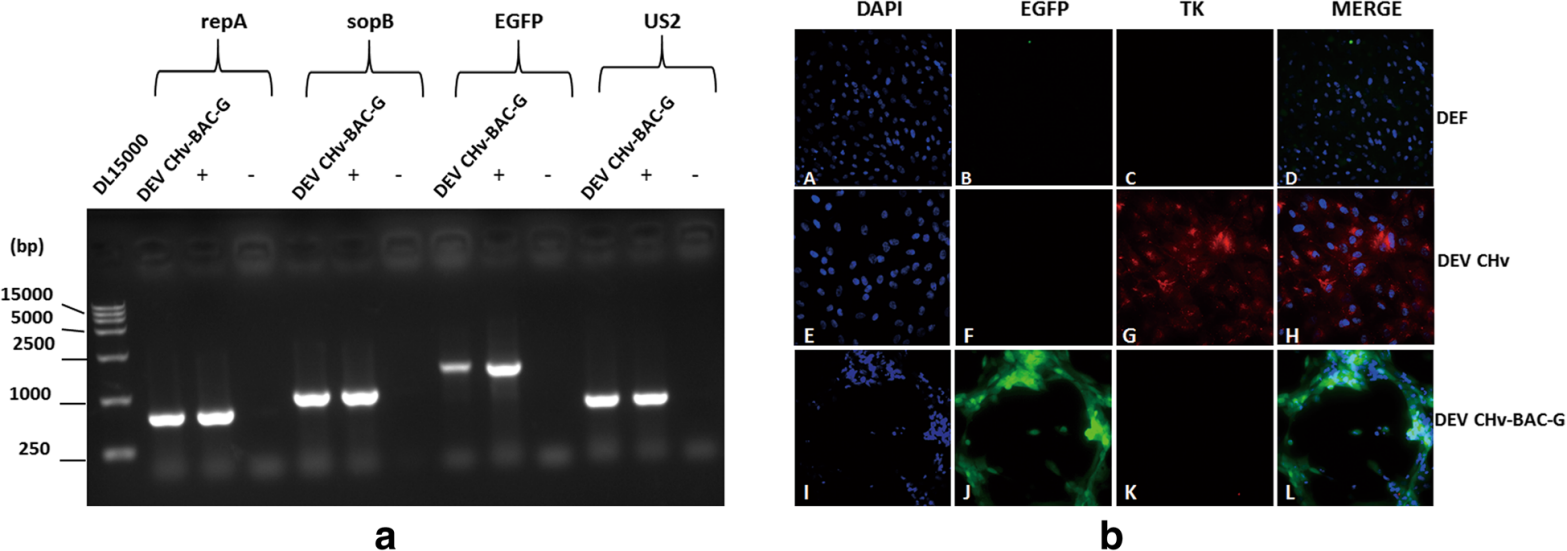
Identification of the rescued DEV CHv-BAC-G in DEF cells. **a** Identification of rescued DEV CHv-BAC-G. repA, sopB, EGFP and US2 were amplified by primers in table 1 and 2 using rescued DEV CHv-BAC-G as template; +:Amplicons of repA, sopB, EGFP and US2 using pBAC-DEV plasmid as template for positive control; -: Amplicons of repA, sopB, EGFP and US2 using DEF cell DNA as template for negative control. **b** Identification of reconstituted DEV CHv-BAC-G by IFA. Polyclonal rabbit anti-TK IgG was taken as primary antibody for detecting of TK protein. *A-D*: mock infected- DEF cells; *I-L*: DEV CHv-BAC-G infected cells; *E-H*: DEV CHv infected cells

An advantage of BAC cloning technology for the manipulation of large DNA viruses is that infectious virus can be reconstituted from the BAC plasmid in host cells. As shown in Fig. 2a, DNA isolated from pBAC-DEV plasmid transfected DEF cells with typical green fluorescence cytopathic effect was used as template for PCR identification. As a result, we found repA, sopB, EGFP and US2 can be detected in reconstitute virus, which indicated the presence of BAC component and DEV genome in reconstituted virus. The expression of TK protein in wild type DEV CHv and derived recombinant DEV CHv-BAC-G infected host cells were recognized by polyclonal rabbit anti-TK in IFA. As shown in Fig. 2b, the presence of green fluorescence in DEV CHv-BAC-G infected cells could attributed to the expression of inserted EGFP gene (Fig. 2b (J)). TK protein was detected only in wild type DEV CHv infected cells (Fig. 2b (G)) but not in DEV CHv-BAC-G and mock infected DEF cells (Fig. 2b (C, K)) indicated the disruption of TK region by transfer vector insertion. Further sequencing results also supported our assertions (data not shown). These data demonstrated that infectious DEV CHv virus could be reconstituted efficiently from BAC plasmid in host cells.

### Characterization of the rescued recombinant viruses in vitro

**Fig. 3:**
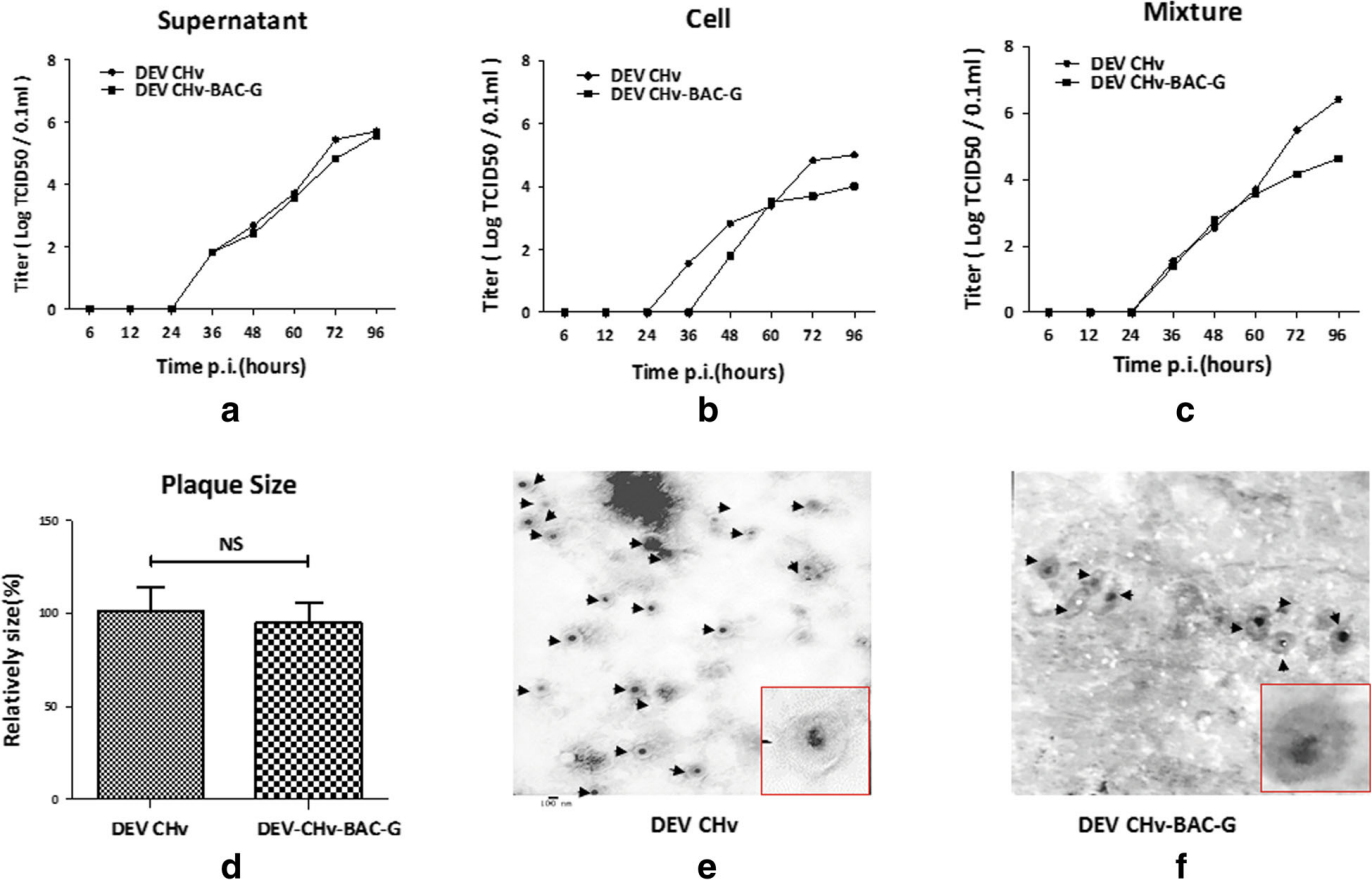
Comparative characterization of DEV CHv-BAC-G and its wild type virus. **a**-**c** Growth curves of wild type DEV CHv and reconstitute virus DEV CHv-BAC-G. DEF cells were infected at an MOI of 0.02, the TCID50 titer of infected supernatant, cells and mixture of cells cultures were titrated at the indicated time points. All titrations were carried out in three independent experiments. **d** Plaque area measurement of DEV CHv and DEV CHv-BAC-G. Means and standard deviations of plaques diameter of each strain were measured with ImageJ software. The Mean of plaque areas of DEV CHv was set at 100%. Standard deviations are shown with the error bar. NS: no significant difference ((*t*-test, *p* > 0.05). **e**, **f** Transmission electron microscopic examination of purified wild type and reconstitute DEV virions. Red box: The virion structure of each virus was scaled up for observation

The recombinant viruses were characterized in vitro by determining replication kinetics, plaque diameters and morphology of virions to detect the effect of BAC vector insertion in TK region on viral replication and assembly. Viral titers were determined by test of TCID50 of viruses. As shown in Fig. 3a-c, the reconstitute virus DEV CHv-BAC-G displayed a slightly lower growth curve during a 96 h period compared to that of wild type DEV CHv due to the insertion of BAC vector into TK gene, but both growth curves exhibited the same trend. There was no increase in the yield of viruses in either supernatant, cell or mixture in the first 24 h. Afterwards, a continuously increasing can be observed in each part of cell cultures. According to the obtained data and references, we speculated that the decreased activity of TK could lead to attenuate virulence without affecting the replication and function of virus [25, 31–33], further studies will be carred out to demonstrate the hypothesis.

In addition, absence of significant difference between plaque diameters of the wild type and recombinant viruses (*t*-test, *p* > 0.05) (Fig. 3d) indicated that the disruption of TK protein didn’t affected the procedures of adsorption, replication, cell-to-cell spread or CPE formation of the reconstituted virus DEV CHv-BAC-G. Transmission electron microscopic examination of the wild type and reconstitute virus infected cells indicated that the structure of them was identical and the functional deficient of TK protein did not affect the structure and assembly of DEV virions (Fig. 3e, f).

### Generation of DEV CHv UL55 deletion mutant and its revertant by RED recombination in E. coli

**Fig. 4:**
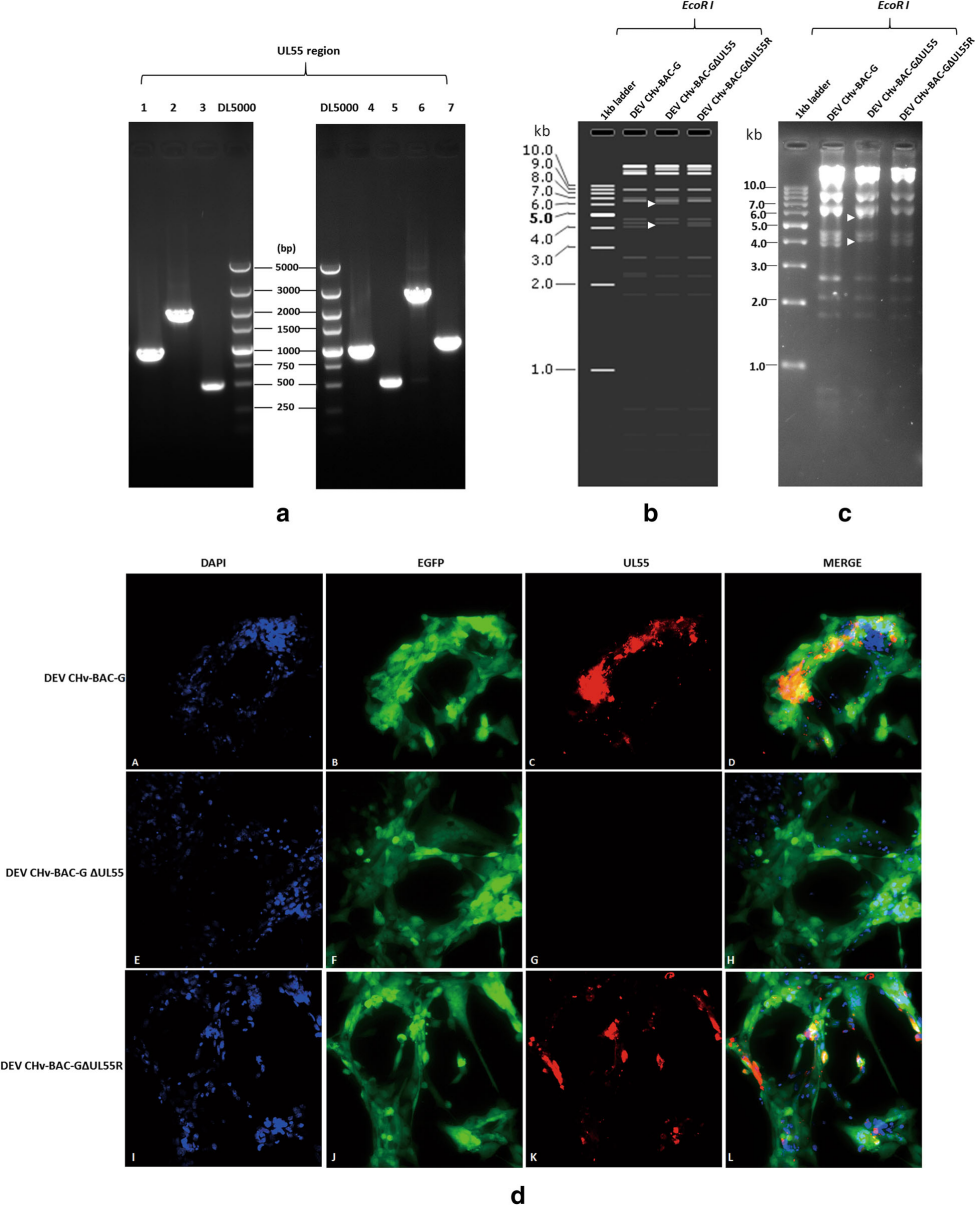
Identification of DEV CHv-BAC-GΔUL55 and its revertant DEV CHv-BAC-GΔUL55R. **a** Identification of DEV CHv-BAC-GΔUL55 and DEV CHv-BAC-GΔUL55R after two rounds of RED recombination. Lane1, 4: The product of UL55 region before RED recombination; Lane 2: The product of UL55 region after 1st round of recombination for constructing UL55 deletion mutant. Lane 3, 5: The product of UL55 region after 2nd round of recombination for constructing UL55 deletion mutant; Lane 6, 7: Identification of 1st and 2nd round of RED recombination for constructing UL55 deletion revertant DEV CHv-BAC-GΔUL55R, respectively. **b**, **c** Restriction fragment length polymorphism (RFLP) analysis of rescued recombinant virus DEV CHv-BAC-G, DEV CHv-BAC-GΔUL55, DEV CHv-BAC-GΔUL55R. (b)(c) Indicated the orientation and real Gel analysis of rescued recombinant virus digested by EcoR I, respectively. △: The different band. **d** Identification of UL55 deletion mutant and revertant by IFA, DEV CHv-BAC-G infected DEF cells were detected as parental virus. Rabbit anti-UL55 IgG were used as primary antibody. *A-D*: DEV CHv-BAC-G infected cells. *E-H*: DEV CHv-BAC-GΔUL55 infected cells. *I-L*: DEV CHv-BAC-GΔUL55R infected cells

Cloning of DEV CHv complete genome as an infectious BAC clone allows arbitrary modification of the viral genomes through different approaches in *E. coli* and reconstitution of the recombinant virus in eukaryotic cells. Based on this newly established platform, the construction of UL55 gene deletion and revertant mutation were carried out by two-step RED recombination for functional study of DEV UL55 gene in DEF cells. As shown in Fig. 4a, the PCR product of 510 bp and 1063 bp was obtained from DEV CHv-BAC-GΔUL55 and DEV CHv-BAC-GΔUL55R mutant. Further RFLP analysis of UL55 mutants and its parental viruses demonstrated the mutagenesis of UL55 gene resulted in loss of a 4-kb *EcoR I* fragment and generation of a new 6-kb fragment (Fig. 4b,c△). Expression of UL55 protein in UL55 deletion and revertant mutants was determined by IFA. From Fig. 4d, we found the UL55 protein could be detected in DEV CHv-BAC-G and DEV CHv-BAC-GΔUL55R infected cells but not in DEV CHv-BAC-GΔUL55 infected cells, while the green fluorescence could be found in parental and mutants infected cells, demonstrated the successfully replication of reconstitute viruses. Thus, the mutation introduced in the DEV CHv-BAC-G plasmid was maintained after reconstitution of mutant and revertant viruses. Rapid generation of infectious UL55 deletion and revertant mutants demonstrated the adaptability of this approach for efficient mutagenesis of DEV CHv genome in *E. coli*.

### Growth properties of UL55 mutant and its parental virus

**Fig. 5:**
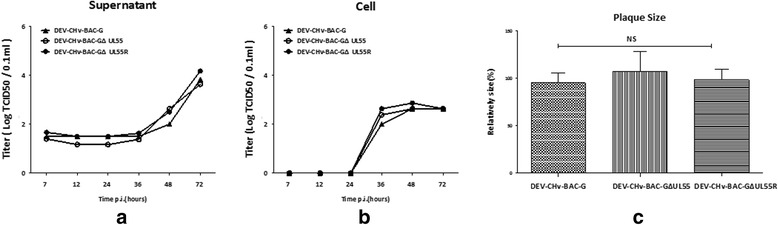
Growth properties of UL55 mutant and its parental virus. **a**, **b** Growth curve of the rescued recombinant DEV CHv-BAC-G, DEV CHv-BAC-GΔUL55 and DEV CHv-BAC-GΔUL55R. DEF cells were infected at an MOI of 0.02, the TCID50 titer of infected superntant and cells of cultures were titrated at the indicated time points. All titrations were carried out in three independent experiments. **c** Plauqe area measurement of DEV CHv-BAC-G,DEV CHv-BAC-GΔUL55 and DEV CHv-BAC-GΔUL55R. Means and standard deviations of plaques diameter of each strain were measured with ImageJ software. Standard deviations are shown with the error bar. NS: no significant difference ((*t*-test, *p* > 0.05)

To determine if the deletion of the UL55 gene from DEV CHv-BAC-G has any effect on the growth properties of recombinant virus, the replication kinetics and plaque diameters were determined and compared to their parental virus. As a result, we found all viruses exhibited comparable growth kinetics on DEF cells in supernatants and cells during a 72 h period. The replication of viruses kept quiescence at the first 24 h after infection, then significant increases were observed during the whole observation time in supernatants while it stopped increasing at 48 h.p.i and slightly dropped after that in cells (Fig. 5a, b). Meanwhile, there was no significant difference in the plaque areas of the parental and recombinant viruses (*t*-test, *p* > 0.05) (Fig. 5c). These results suggested that the deletion of UL55 gene from DEV genome has no effect on the growth properties of DEV CHv and UL55 gene is dispensable for DEV replication.

### The effect of UL55 protein on the intracellular distribution of UL26.5 protein

**Fig. 6:**
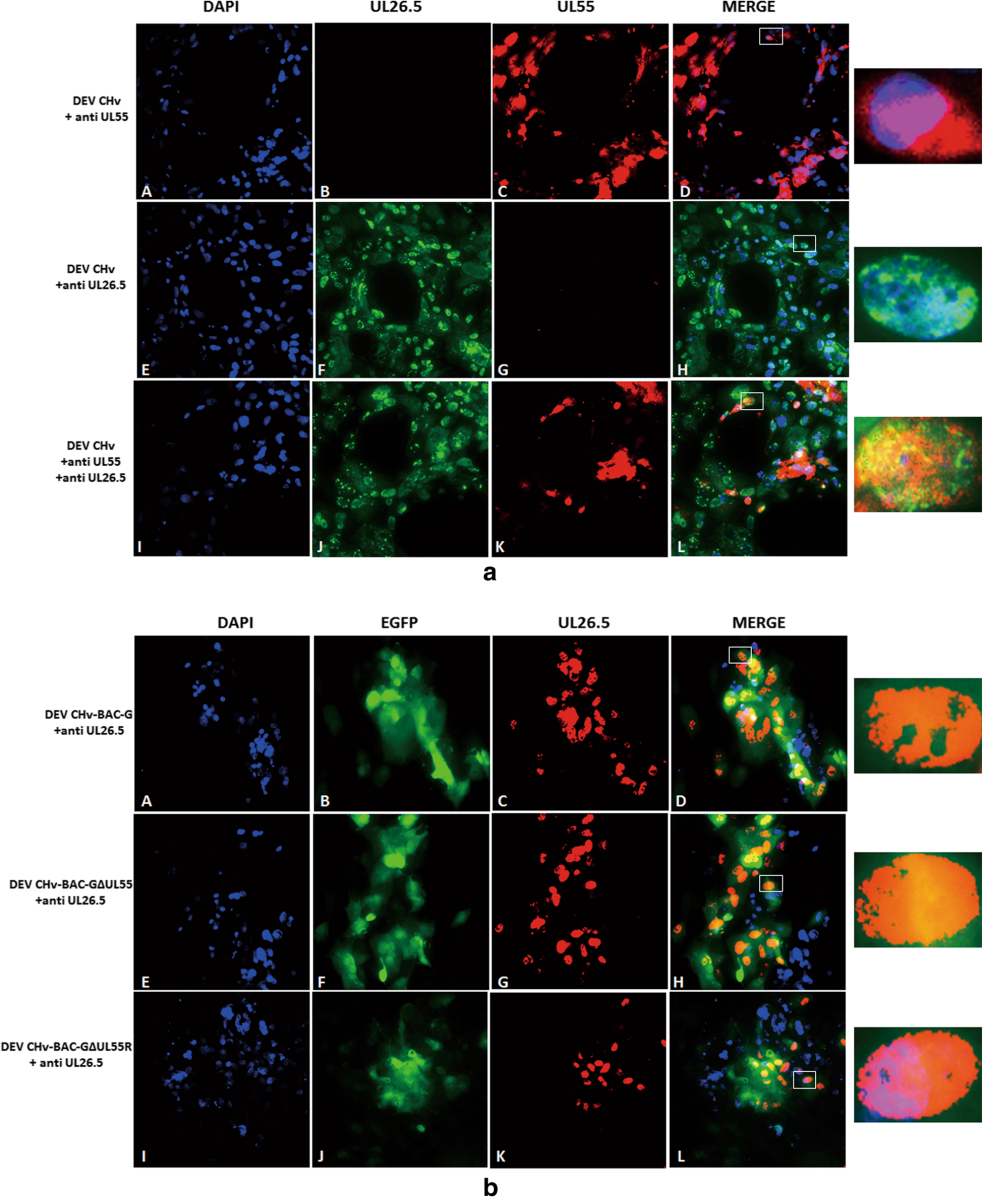
The effect of UL55 protein on the intracellular distribution of UL26.5 protein. **a** Intracellular distribution analysis of UL55 and UL26.5 protein in DEV CHv infected cells. *A-D*: Using anti mouse UL55 IgG as primary antibody for detecting the intracellular distribution of UL55 protein. *E-H*: Using anti rabbit UL26.5 IgG as primary antibody for detecting the intracellular distribution of UL26.5 protein; *I-L*: Using anti mouse UL55 IgG and anti-rabbit UL26.5 IgG together as primary antibody for colocalization analysis of UL55 and UL26.5 protein. **b** Intracellular distribution of UL26.5 protein in the absence of UL55 gene. *A-D*: Using anti rabbit UL26.5 IgG as primary antibody for distribution analysis of UL26.5 protein in DEV CHv-BAC-G infected cells; *E-H*: Using anti rabbit UL26.5 IgG as primary antibody for distribution analysis of UL26.5 protein in DEV CHv-BAC-GΔUL55 infected cells; *I-L*: Using anti rabbit UL26.5 IgG as primary antibody for distribution analysis of UL26.5 protein in DEV CHv-BAC-GΔUL55R infected cell

The distribution of UL26.5 protein in DEF cells with presence or absence of UL55 protein was determined by indirect immunofluorescence experiments. As shown in Fig. 6a(A-D), the UL55 protein was mainly distributed in bright fluorescent granules in cytoplasm near the periphery of the nucleus at 60 h p.i., and a small amount of it distributed in the nucleus, while the UL26.5 protein was distributed in widespread speckled structures in the nuclei of infected cells (Fig. 6a(E-H)). Co-localization analysis showed that the UL55 protein abutted on and partially overlapped the UL26.5 protein, and the abutting feature was the predominant. The localization features of UL55 and UL26.5 protein were as same as that of HSV-2 UL55 protein.

UL55 deletion mutant was used to infect DEF cells to detect the distribution of UL26.5 protein in the absence of UL55 protein. Its parental and revertant viruses were infected as controls. In Fig. 6b(C, G, K), the red fluorescence speckled structures were observed in the nucleus of DEV CHv-BAC-GΔUL55 infected cells, which were also observed in its parental and revertant virus infected cells. Green fluorescence was observed in all viruses infected cells indicated the successfully replication of viruses. These results demonstrated that the distribution of UL26.5 protein encoded by virus lacking UL55 protein were identical to that of wild type virus. In spite of the abutting and overlapping features of UL26.5 and UL55 protein in DEV infected cells, the distribution of UL26.5 protein is independent of UL55 protein.

## Discussion

As full genome sequence and organization of DEV have been published to date, specific gene functions could be characterized. To our knowledge, however, the specific characteristic and function of the UL55 protein in DEV CHv are still unknown due to the limited technology of genome manipulation in host eukaryotic cells [15]. Recent advance in biologic technologies bring in many genome editing approach including the most popular CRISPR/Cas9 system. Based on this, scientists have been successfully knocking out protein-coding genes in several model organisms [34]. It is now become an important approach for understanding gene functions and for engineering genetic regulatory systems. However, the successful rate of gene knock out/down is not 100% because of its limitations. The non-specificity of guide RNA target sequence and the uncertainty of DNA damage repair both may result unknown mutations in the other locus of genome (off-target effect), especially in organisms with large genomes [35]. Compared to this, a full length genomic cloning technology based on BAC allowing the recombinants to be reconstituted as an infectious virus in host cells after mutagenesis in *E. coli* become an optimal alternative approach for herpes virus genome manipulation. In this strategy, large herpes virus genomes can be manipulated accurately and rapidly in *E. coli* without unwanted recombination events or rearrangements due to the only homologous double-strand ends will be used as a substrate [36, 37]. The stability of the recombinant BAC clone in *E. coli* can be maintained because of the strict replicons in BAC mini-F sequence. On basis of that, infectious BAC clone is considered as a preferred large-insert cloning system for genomic analysis and gene discovery in herpes virus, and undoubtedly contributed a lot to investigations of virus life cycles, vaccines, gene functions and pathogenesis [37–39]. Thus, we firstly constructed an infectious BAC clone of the Duck Enteritis Virus Chinese virulent strain (DEV CHv) for investigation of UL55 gene function in infected DEF cells in this paper. We believe outcomes will provide some clues for understanding mechanisms of DEV life cycle and that involved in DEV pathogenicity.

Based on the established infectious DEV CHv BAC clone, mutagenesis of UL55 gene was generated by two-step RED recombination for testing the amenability of this platform for DEV CHv gene study. The major advantage of the Red recombination system is that only short homologous sequences of 30 to 50 bp are required for the recombination to proceed. For the recombination in bacteria, components of the Red or RecE/T recombination system can be delivered *in trans* by plasmids such as pKD46 that allow inducible expression of Alpha, Beta and Gam. Once the mutagenesis procedure is completed, pKD46 can be cured from bacteria by its temperature-sensitive replication mechanism. Furthermore, the unwanted selection markers introduced by 1st round RED recombination can be excised from the recognition sites by Flp recombinase [30, 37]. Besides, compared to the popular en passant protocol based on BAC maintained in GS1783, the traditional two-step recombination methods needs several help plasmids was much more accurate in the first round of mutagenesis. Thus, the combination of DEV infectious BAC clone system and RED recombination or other mutagenesis technologies allows easily generation of a variety of different modifications of DEV CHv. To exclude the random mutation of UL55 gene in genetics procedures, the deletion of UL55 gene was repaired by revertant mutation using RED recombination. The subsequent transfection into DEF cells demonstrated that UL55 gene is dispensable for DEV CHv replication and the UL55 deletion mutants exhibit identical growth characteristics to its parental and revertant virus in vitro*.*

Study of HSV-2 UL55 gene suggested that UL55 protein abutted on and partially overlapped with the UL26.5 gene encoded capsid protein ICP35 in intracellular [20]. To our knowledge, virus relies on host synthetic machine to complete its replication, biological functions of mature proteins must occur at the specific locations in cells [40]. The intracellular compartmentation distribution can affect the folding, aggregation and posttranscriptional modification process of protein and further effects on function of cells [41]. The abutting and overlapping distribution features of UL55 and UL26.5 protein in DEV CHv infected cells revealed functional correlations of UL55 and UL26.5 protein in virus replication. The co-localization features could be explained as the UL26.5 encoded protein is involved in viral capsid assembly as a scaffold protein. The assembled capsid protein gets transferred to the periphery of nuclei close to nuclear membrane for releasing into cytoplasm and subsequently the nearby UL55 included assemblons will accomplish the viral assembly in cytoplasm subsequently. The abutting feature in intracellular was aimed to execute the viral package function of UL55 protein. The explanation for the overlapping distribution of UL55 and UL26.5 protein could be attributed to the evacuation and degradation of UL26.5 protein after the capsid synthesis. The resulted aggregation of falling off UL26.5 protein from inner capsid structure in cytoplasm and nucleus may be detected by anti UL26.5 IgG and overlapped with fluorescence produced by the widely distributed UL55 protein in cytoplasm. Further distribution analysis of UL26.5 protein in the UL55 negative mutant infected DEF cells revealed that the absence of UL55 protein didn’t affect the distribution and function of UL26.5. Herein, we proposed a hypothesis that the assembly process of capsid constituted by UL26.5 and other component part may involves in multiple proteins. DEV UL55 protein plays a role in viral assembly, but it can be substituted. Further work is being carried out to testify the possibility of UL55 gene locus as an exogenous gene insertion site for developing vectored vaccine and virus attenuation. We believe the obtained data will supply some important information for virus pathogenesis research and vaccine development.

## Conclusions

In conclusion, we have successfully developed an infectious BAC clone of lethal clinical isolate DEV CHv for the first time. The generated UL55 gene mutant demonstrated this platform would be a very useful tool for DEV gene manipulation. DEV UL55 gene is dispensable in virus replication and UL26.5 distribution in virus infected cells. Experiments are now in progress to testify the possibility of UL55 gene locus as an exogenous gene insertion site for developing vectored vaccine after attenuation. We believe the obtained data will supply some important information for virus pathogenesis and vaccine development as well.

## Additional files

Additional file 1: Figure S1. Schematic illustration of the strategy used to construct transfer vector pUC18/EGFP-TKAB-BAC11. The number in circle indicated different cloning steps. ❶: Cloning of EGFP into pUC18 for constructing pUC18/EGFP. ❷❸: TKA and TKB were amplified from DEV CHv and subsequently cloned into pUC18-EGFP to generate pUC18/EGFP-TKAB. ❹: Linearized pUC18/EGFP-TKAB and BAC Mini-F sequence donor pBeloBAC11 were obtained by *Sph I* digestion. ❺: Transfer vector pUC18/EGFP-TKAB-BAC11 harboring the homologous regions of TK insertion site, mini-F sequence of BAC and a cellular screening marker EGFP was generated after ligation. (PDF 534 kb)

Additional file 2: Figure S2. Schematic illustration of the strategy used to construct infectious DEV CHv BAC clone, UL55 deletion mutant and it’s revertant. (a) Schematic presentation of the organization of the entire genome of the wild type DEV CHv strain. The terminal and internal repeat sequence of genome, TK and UL55 ORFs were shown. The transfer vector used for homologous recombination in host cells contains mini-F sequence of BAC, enhanced green fluorescent protein (EGFP), two copies of the direct orientation 34-bp Loxp and homologous arms flanking regions of TK insertion site.* : the insertion site of transfer vector in TK region. (b) Schematic presentation of the reconstituted DEV CHv-BAC-G infectious clone after insertion of the transfer vector pUC18/EGFP-TKAB-BAC11. A kanamycin resistance cassette flanked by FRT sites and 50 bp homology arms of UL55 gene was used to replace UL55 gene in the 1st round of RED recombination induced by pKD46. Another temperature sensitive plasmid Pcp20 was introduced into system for elimination of KanR in the 2nd round of RED recombination by utilizing the Flplase. (c) Schematic presentation of the resulted UL55 deletion mutant DEV CHv-BAC-GΔUL55. A linear fragment contains UL55 gene and KanR cassette flanked by homology arms of UL55 gene was used for constructing UL55 deletion revertant mutant by two step RED recombination as previously described. (d) Schematic presentation of the resulted UL55 deletion revertant mutant DEV CHv-BAC-GΔUL55R after two rounds of RED recombination. (PDF 755 kb)

## Abbreviations

BAC: Bacteria artificial chromosome
BoHv-1: Bovine herpesvirus 1
CHv: Chinese virulent strain
CPE: Cytopathic effect
DEF: Duck embryo fibroblast
DEV: Duck enteritis virus
DP: Duck plague
DVE: Duck viral enteritis
EBV: Epstein–barr virus
EGFP: Enhanced green fluorescent protein
EHV-1: Equine herpesvirus 1
GPCMV: Guinea pig cytomegalovirus
h.p.i: Hours post infection
HCMV: Human cytomegalovirus
HSV: Herpes simplex virus
HVT: Turkey herpesvirus
IFA: Indirect immunofluorescence analysis
KanR: Kanamycin-resistance
KSHV: Kaposi’s sarcoma herpesvirus
MDV: Marek’s disease virus
MEM: Minimal essential medium
MHV-68: Murine gammaherpesvirus 68
MOI: Multiplicity of infection
NCS: New calf serum
P/S: Penicillin, streptomycin
PRV: Pseudorabies virus
SDS: Sodium dodecyl sulfate
VZV: Varicella zoster virus
