# Supplementary figures and images for: Preliminary study of the UL55 gene based on infectious Chinese virulent duck enteritis virus bacterial artificial chromosome clone
Source: Virol J. 2017 Apr 13;14:78. doi: 10.1186/s12985-017-0748-y (PMC5390382; doi:10.1186/s12985-017-0748-y)

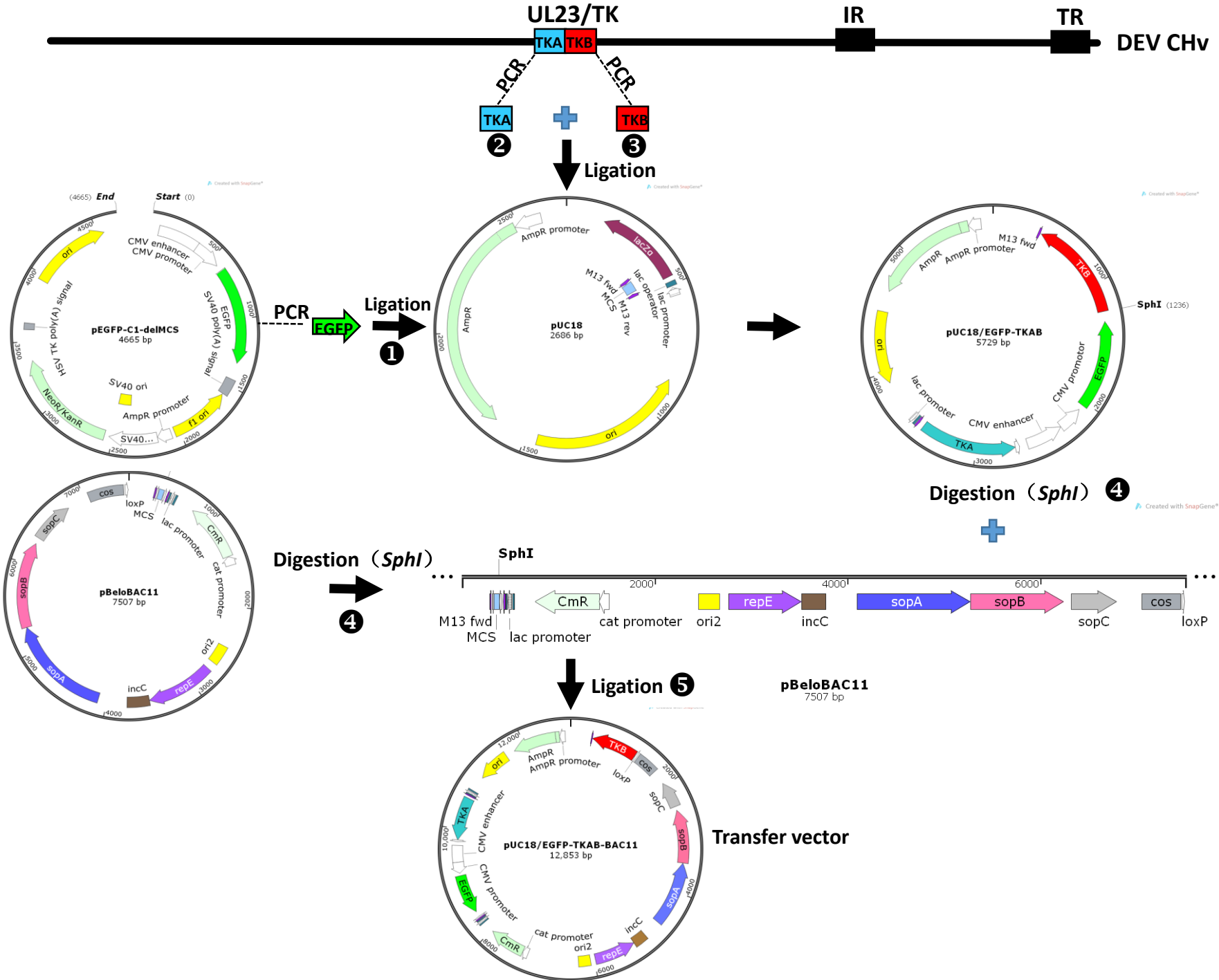

Supplement: Supplementary file 1 — Schematic illustration of the strategy used to construct transfer vector pUC18/EGFP-TKAB-BAC11. The number in circle indicated different cloning steps. ❶: Cloning of EGFP into pUC18 for constructing pUC18/EGFP. ❷❸: TKA and TKB were amplified from DEV CHv and subsequently cloned into pUC18-EGFP to generate pUC18/EGFP-TKAB. ❹: Linearized pUC18/EGFP-TKAB and BAC Mini-F sequence donor pBeloBAC11 were obtained by Sph I digestion. ❺: Transfer vector pUC18/EGFP-TKAB-BAC11 harboring the homologous regions of TK insertion site, mini-F sequence of BAC and a cellular screening marker EGFP was generated after ligation. (PDF 534 kb) [file 12985_2017_748_MOESM1_ESM.pdf]
