# Supplementary material for: Preliminary study of the UL55 gene based on infectious Chinese virulent duck enteritis virus bacterial artificial chromosome clone
Source: Virol J. 2017 Apr 13;14:78. doi: 10.1186/s12985-017-0748-y (PMC5390382; doi:10.1186/s12985-017-0748-y)
Supplement: Supplementary file 2 — Schematic illustration of the strategy used to construct infectious DEV CHv BAC clone, UL55 deletion mutant and it’s revertant. (a) Schematic presentation of the organization of the entire genome of the wild type DEV CHv strain. The terminal and internal repeat sequence of genome, TK and UL55 ORFs were shown. The transfer vector used for homologous recombination in host cells contains mini-F sequence of BAC, enhanced green fluorescent protein (EGFP), two copies of the direct orientation 34-bp Loxp and homologous arms flanking regions of TK insertion site.* : the insertion site of transfer vector in TK region. (b) Schematic presentation of the reconstituted DEV CHv-BAC-G infectious clone after insertion of the transfer vector pUC18/EGFP-TKAB-BAC11. A kanamycin resistance cassette flanked by FRT sites and 50 bp homology arms of UL55 gene was used to replace UL55 gene in the 1st round of RED recombination induced by pKD46. Another temperature sensitive plasmid Pcp20 was introduced into system for elimination of KanR in the 2nd round of RED recombination by utilizing the Flplase. (c) Schematic presentation of the resulted UL55 deletion mutant DEV CHv-BAC-GΔUL55. A linear fragment contains UL55 gene and KanR cassette flanked by homology arms of UL55 gene was used for constructing UL55 deletion revertant mutant by two step RED recombination as previously described. (d) Schematic presentation of the resulted UL55 deletion revertant mutant DEV CHv-BAC-GΔUL55R after two rounds of RED recombination. (PDF 755 kb) [file 12985_2017_748_MOESM2_ESM.pdf]

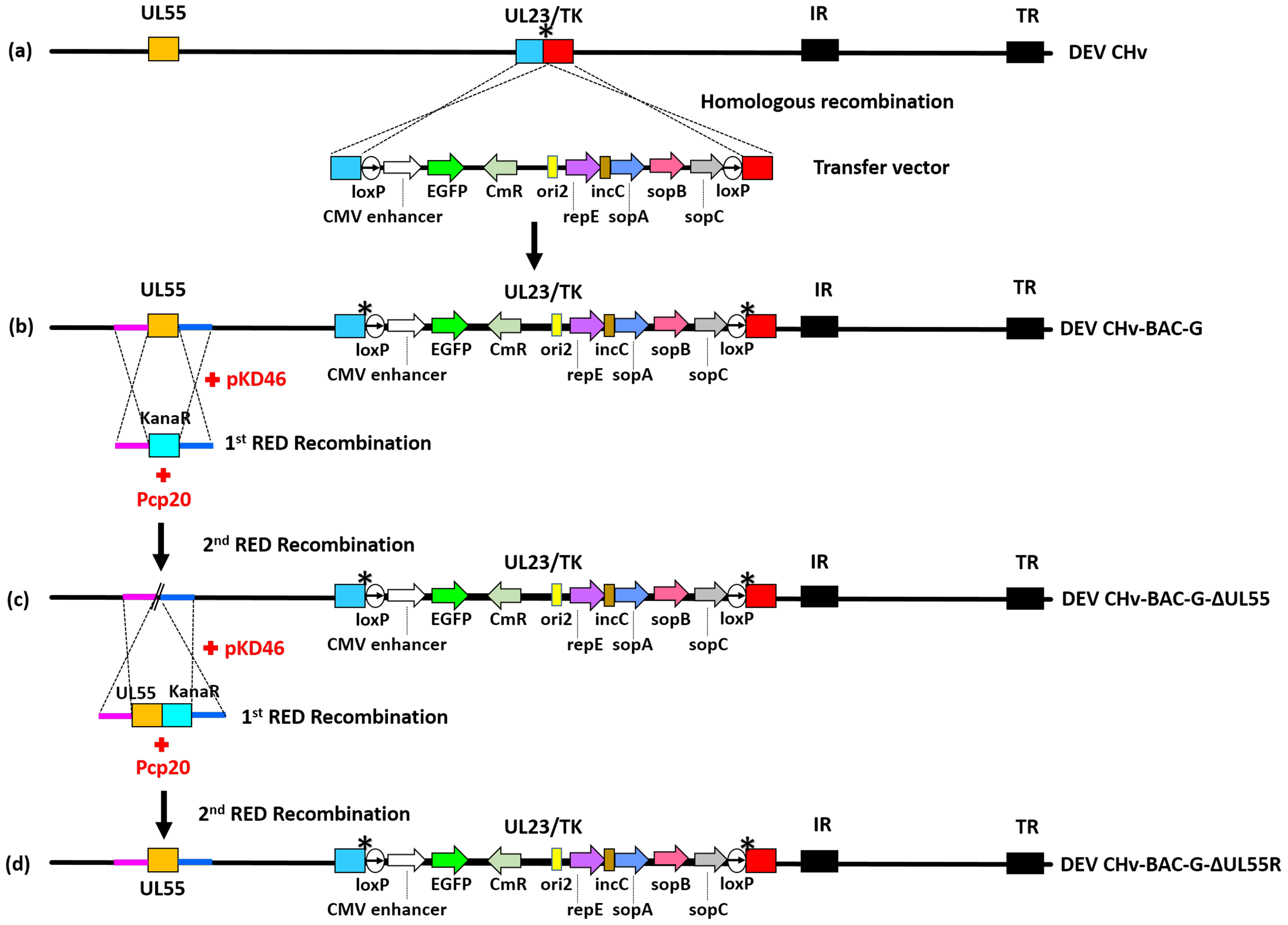

(a)
UL55
UL23/TK
IR
TR
DEV CHv
Homologous recombination
Transfer vector
loxP
CMV enhancer
EGFP
CmR
ori2
repE
incC
sopA
sopB
sopC
loxP
(b)
UL55
UL23/TK
IR
TR
DEV CHv-BAC-G
pKD46
KanaR
1st RED Recombination
Pcp20
2nd RED Recombination
(c)
UL23/TK
IR
TR
DEV CHv-BAC-G-ΔUL55
pKD46
UL55
KanaR
1st RED Recombination
Pcp20
2nd RED Recombination
(d)
UL55
UL23/TK
IR
TR
DEV CHv-BAC-G-ΔUL55R
